# Exposure to environmental tobacco smoke and prevalence of asthma among adolescents in a middle eastern country

**DOI:** 10.1186/s12889-020-09245-9

**Published:** 2020-08-08

**Authors:** Hussain Booalayan, Mosa Abdualrasool, Saad Al-Shanfari, Abdulwahab Boujarwa, Abdullah Al-Mukaimi, Omar Alkandery, Saeed Akhtar

**Affiliations:** Department of Community Medicine and Behavioral Sciences, Faculty of Medicine, Kuwait University, PO Box 24923, 13110 Safat, Kuwait

**Keywords:** Environmental tobacco smoke, Asthma, Adolescents, Prevalence ratio, Log-binomial model

## Abstract

**Background:**

There is increasing evidence linking environmental tobacco smoke (ETS) exposure at homes to the development of asthma among adolescents. Few studies have addressed this issue in the Middle Eastern countries including Kuwait. Therefore, this cross-sectional study assessed the prevalence of ETS exposure at home, prevalence of asthma and other respiratory conditions and examined the ETS exposure at home and personal tobacco smoking as risk factors for self-reported asthma among high-school students in Kuwait.

**Methods:**

In this cross-sectional study, we enrolled participants from nine high-schools of Hawally Governorate of Kuwait during October 2015. We adapted a previously validated self-administered questionnaire for data collection. Prevalence of self-reported asthma and ETS exposure (≥ 1 smoker at home vs. none) were computed. The association between exposures of interest and self-reported asthma status was examined using a multivariable log-binomial regression model.

**Results:**

Of 800 enrolled participants, 746 (92.2%) consented and completed the questionnaire. The participants with mean (SD) age of 16.8 (0.68) years were predominantly Kuwaiti (74.8%) and female (50.1%). The prevalence of ETS exposure at home and personal current smoking was 54 and 12.4% respectively. Self-reported asthma prevalence was 20.5%. Furthermore, the prevalence of physician-diagnosed asthma, wheezing during the last 12 months and wheezing ‘ever’ was 16.4, 20.1 and 26.2%, respectively. Fitted multivariable log-binomial regression model revealed that compared with the non-asthmatic, participants with self-reported asthma tended to be current smokers (adjusted prevalence ratio (adjusted PR) = 1.82; 95% CI: 1.30–2.56; *p* = 0.001) or have had ETS exposure at home (adjusted PR = 1.64; 95% CI: 1.21–2.23; *p* = 0.002).

**Conclusions:**

We recorded a high prevalence of ETS exposure at home, high prevalence of self-reported asthma and identified ETS exposure at home and being a current smoker as strong risk factors for self-reported asthma among adolescents. Voluntary household smoking bans may substantially minimize the ETS exposure among adolescents. Additionally, such restriction may inculcate an antismoking attitude and prevent smoking initiation among adolescents. Such efforts may bring about reduction in ETS exposure and associated asthma risk and other smoking-related morbidities in this and other similar settings.

## Background

Asthma is one of the most important diseases of childhood, causing substantial morbidity [1], increases the hospital admission rates and primary care contacts [2]. Data from the ISAAC (International Study of Asthma and Allergies in Childhood) Phase-III study have shown an estimated global asthma prevalence among adolescents as 14.1% with an average annual increase of 0.28% from previous estimate during ISAAC Phase II study [3]. Furthermore, the results of ISAAC Phase -III study showed that virtually all the countries reported increases in the lifetime asthma prevalence between phases I and III. Globally, a wide variability (range: 2.1–32.2%) in the asthma prevalence has been recorded, not only between the regions and countries but also within the same country suggesting a crucial role of local environmental characteristics [4]. This prevalence of asthma was particularly high in English speaking countries and Latin America [5]. Also, the highest 12-month prevalence of asthma was recorded in the UK, Australia, New Zealand, and Republic of Ireland, followed by North, Central, and South America; the lowest prevalence was reported from several Eastern European countries, Indonesia, Greece, China, Taiwan, Uzbekistan, India, and Ethiopia [6]. In Saudi Arabia, lately a ISAAC study reported the prevalence of physician-diagnosed asthma among adolescents as 19.6% [7]. In Kuwait, among 13–14 years adolescents, the estimated physician-diagnosed prevalence of asthma in ISAAC Phase I (16.8%) [8], and ISAAC III (15.6%) [9], almost remained unchanged. However, little pusblished data on the recent status of asthma among adolescents in the Middle-Eastern countries including Kuwait are available.

There is a considerable evidence that exposure to environmental tobacco smoke (ETS) among children and adolescents has several ill-health consequences including an elevated risk of sudden infant death syndrome, reduced lung growth, early development of cardiovascular diseases, atopic dermatitis, increased susceptibility to respiratory infection and childhood asthma onset [10]. The effect of ETS exposure on respiratory health has been of interest for many years [11]. The available epidemiological evidence on the relationship between ETS exposure and childhood asthma is largely based on the studies carried out in the Western countries [12]. Little published data on the link between ETS exposure at home and asthma among adolescents are available from the Middle Eastern countries. In Kuwaiti population, tobacco consumption is practiced by different modes including cigarettes (34.4%), waterpipe (63.0%), both cigarettes and waterpipe (7.9%) [13]. Furthermore, smoking is much more common among men (32.4%) than women (1.5%) [13]. It is estimated that one-third of the Kuwaiti population are smokers [14], and 54% of the adolescents get exposed to ETS at homes [15]. Another recent study from Kuwait reported high prevalence of ETS exposure at home among the students enrolled in the middle schools (45.8%) and the high schools (51.6%) [16]. To minimize ETS exposure in Kuwait, the public health authorities regularly conduct educational campaigns through electronic and print media and has banned the use of tobacco products in public places, such as malls, public offices, public gardens, etc. However, the impact evaluation of such efforts on the burden of ETS exposure related allergic conditions including asthma among children is still awaited. Therefore, the objectives of this cross-sectional study were to i) assess the prevalence of self-reported asthma, and ii) examine the association between ETS exposure at home and self-reported asthma among adolescents enrolled in various high schools in Kuwait.

## Methods

### Study design, setting and population

The study design, setting, and population have been previously described elsewhere [17], and briefly outlined here. During October 2015, we conducted a cross-sectional study among high-school students in Hawally - one of the six Governorates of Kuwait to i) assess the prevalence of ETS exposure at home, ii) assess the prevalence of various allergic conditions including asthma and iii) evaluate the association between ETS exposure at-home and self-reported asthma. The foremost contemplation in selecting this study population was their high anticipated frequency and ETS exposure period and expected high prevalence of asthma and other allergic conditions. From the Ministry of Education’s website, we obtained a list of schools located in Hawally Governorate. From this list of ten boys’ schools, nine girls’ schools in public-sector and forty five private schools with co-education system, nine schools were selected as a sample of convenience, which included public-sector schools for boys (3) and girls (3) and private-sector schools with co-education system (3) [17].

### Questionnaire

A structured and self-administered questionnaire was developed in English for the collection of data on socio-demographic variables, smoking status, and exposure to tobacco smoke at home and in public areas as described elsewhere [17]. For the diagnosis of asthma, the standardized international study of asthma and allergies in childhood (ISAAC) core questionnaire was used [18]. In this study, a respondent was regarded as an asthmatic, if during the past 12 months, he or she reported to have had four or more episodes of wheezing or one or more episodes of wheezing with the use of an inhaler (i.e. self-reported asthma) [18]. We also asked whether the respondent was ever diagnosed as asthmatic by a physician (physician-diagnosed asthma). As noted above, in this study, self-reported asthma was defined based on ISAAC criteria [18], and used as an outcome variable for risk factors analysis. The questionnaire was developed in English and the final version was also translated in Arabic for actual use. The questionnaire was pre-tested on 20 high school students and modifications were made as needed. The questionnaire comprised 21 questions and took on average five minutes for its completion [17].

### Data collection

We planned to enroll around 100 students from various sections of 11th and 12th grades of the selected schools. For this goal, we sought the help of the relevant section in-charge teacher in having the questionnaire filled-in by the students when the class was over. The teachers explained the study objctives to the students, and they were additionally informed that their study participation was voluntary. We used the same data collection procedure both for public and private-sector schools. For this cross-sectional study, we estimated a sample size of 827 students to assess the prevalence of self-reported asthma at 95% confidence level (1-α) with 2.5% bound on error of estimation assuming a prevalence of self-reported asthma as 16% in our target population [8, 9]. To account for any potential refusals, final sample size was inflated to 850 students. The study protocol was approved by the Kuwait University, Health Sciences Center’s Ethics Committee for Students’ Research.

### Statistical analysis

To characterize the study sample, descriptive statistics including mean (standard deviation (SD)) for continuous variables and frequencies (%) for nominal variables were computed. The statistical significance of the association between each of the independent variables and self-reported asthma status was evaluated using Pearson’s chi-squared test. Since odds ratio tends to overestimate the magnitude of association between the independent variables and a common outcome, we used the crude and adjusted prevalence ratio (PR) as a measure of association between the independent variables and self-reported asthma status. Univariable log-binomial regression model was used to quantify the magnitude of unadjusted association of each the categorical variables with self-reported asthma status. The variables significantly (*p* ≤ 0.150) associated with self-reported asthma on unadjusted analysis were considered for inclusion in multivariable log-binomial regression model. Backward stepwise procedure was adapted to arrive at the final multivariable log-binomial regression model. The variables independently and significantly (*p* <  0.05) related with self-reported asthma status were retained in the final multivariable log-binomial regression model. Furthermore, regardless of the statistical significance, age, gender, and total family income (Kuwaiti Dinars/ month) were included in the final model to adjust for their confounding effects. Adjusted PRs and their 95% confidence intervals (CI) were used for the interpretation of the results.

## Results

### Descriptive statistics, prevalence of asthma

Of 800 adolescents invited for enrollment in the study, 746 (93.3%) from nine schools consented and filled-in the study questionnaire. Nine schools included six public-sector (3 each for boys and girls) and three private-sector schools with co-education system. Non-respondents’ age and gender distributions were almost similar to those of the respondents. The mean (SD) age of the adolescents was 16.78 (0.68) years. The adolescents predominantly were Kuwaiti (74.8%), female (50.1%), and 56.1% came from the homes having family income more than a thousand Kuwaiti Dinars (≈ 3210 US $) per month. Of the respondents, 91 (12.4%) were current regular smokers, substantially more among males (22.1%) than females (2.7%). Of the smokers, 55 (62%) have been smoking for > 2 years. Of the adolescents, 398 (54%) had ETS exposure at home (i.e. had ≥1 smokers at home). Moreover, 52.3% of the adolescents reportedly had ETS exposure at public places for ≥3 h a week. The prevalence of self-reported asthma was 20.5%. Furthermore, the prevalence of physician-diagnosed asthma, wheezing during last 12 months and wheezing ‘ever’ was 16.4, 20.1 and 26.2%, respectively (Table 1).

**Table 1:** Socio-demographic characteristics, cigarette smoking status and exposure to environmental tobacco smoke at home and prevalence of self-reported asthma^a^ among participants. October 2015 (*N* = 746)

Characteristics	n	%
Type of school		
Government	594	79.0
Private	157	21.0
Age, mean (SD)	16.78 (0.68)	
Gender		
Male	372	49.9
Female	374	50.1
Nationality		
Kuwaiti	552	74.8
Non-Kuwaiti	186	25.2
Income (Kuwaiti Dinars)		
< 500	32	4.7
500–1000	126	18.3
1001–1500	144	20.9
1501–2000	105	15.3
> 2000	281	40.8
Respondent smoking status (cigarettes per day)		
None	643	87.6
Less than 10	45	6.1
10 or More	46	6.3
Smoking duration		
< 1 year	13	14.6
Between 1 and 2 years	21	23.6
> 2 years	55	61.8
Smokers at home		
None	338	45.9
One or more	398	54.1
ETS exposure at public places (hours per week)		
< 3	336	47.7
3 to 6	205	29.1
6 to 9	75	10.6
> 9	89	12.6
Wheezing ever		
Yes	197	26.2
No	554	73.8
Wheezing in the last 12 months		
Yes	151	20.1
No	600	79.9
Physician-diagnosed asthma		
Yes	123	16.4
No	628	83.6
Self-reported asthma status		
Yes	154	20.5
No	597	79.5

^a^ Diagnosed as per ISAAC criteria

### Univariable and multivariable log-binomial regression models

On univariable analyses, the variables significantly related with self-reported asthma status were adolescent’s personal smoking status (PR = 1.96; CI: 1.43–2.69; *p* <  0.001), ETS exposure at home (presence of ≥1 smokers at home) (PR = 1.75; CI: 1.29–2.37; *p* <  0.001), and ETS exposure at public places (PR = 1.46; CI: 1.09–1.96; *p* = 0.013). Gender, nationality, and total monthly family income (Kuwaiti dinars) were not statistically significantly related with self-reported asthma status in unadjusted analyses (Table 2). A multivariable model revealed that compared with non-asthmatic, adolescents with self-reported asthma tended to be current smokers (adjusted PR = 1.82; 95% CI: 1.30–2.56; *p* = 0.001) or have had ETS exposure at home (adjusted PR = 1.64; 95% CI: 1.21–2.23; *p* = 0.002) after adjusting for the effects of age, gender and total family monthly income (Table 3).

**Table 2:** Univariable analysis of adolescents’ characteristics associated with their self-reported asthma status in Kuwait: A cross-sectional study, October 2015 (n = 746)

Characteristics	Total n	Self-reported asthma^a^ (yes vs. no) n (%)	Unadjusted prevalence ratio (95% CI)	*p-*value^b^
Gender				0.163
Female	374	69 (18.4)	1.00	
Male	372	85 (22.8)	1.24 (0.93–1.64)	
Nationality				0.865
Non-Kuwaiti	186	37 (19.9)	1.00	
Kuwaiti	552	115 (20.8)	1.05 (0.76–1.47)	
Income (Kuwaiti Dinars)				0.974^c^
< 500	32	5 (15.6)	1.00	
500–1000	126	28 (22.2)	1.42 (0.60–3.39)	
1001–1500	144	33 (22.9)	1.38 (0.58–3.30)	
1501–2000	105	19 (18.1)	1.34 (0.54–3.33)	
> 2000	281	64 (22.8)	1.69 (0.73–3.92)	
Respondent’s current smoking status				< 0.001
No	643	119 (18.5)	1.00	
Yes	91	33 (36.3)	1.96 (1.43–2.69)	
Number of smokers at home				< 0.001
None	338	50 (14.8)	1.00	
One or more	398	103 (25.9)	1.75 (1.29–2.37)	
ETS exposure at public places (hours per week)				0.013
< 3	336	58 (17.3)	1.00	
≥ 3	369	93 (25.2)	1.46 (1.09–1.96)	

*ETS* Environmental Tobacco Smoke

^a^Diagnosed as per ISAAC criteria

^b^*p*-values are for Yate’s corrected Chi-squared statistic unless stated otherwise,

^c^*p*-value for Chi-squared statistic for trend

**Table 3:** Multivariable log-binomial regression model^a^ of the variables associated with the respondent’s self-reported asthma status in Kuwait: A cross-sectional study, October 2015

Variables	Self-reported asthma status (yes vs. no) ^b^	
	Adjusted prevalence ratio (95% confidence interval)	*p-value*
Respondent current smoking status (yes vs. no)	1.82 (1.30–2.56)	0.001
Number of smokers at home (one or more vs. none)	1.64 (1.21–2.23)	0.002

^a^ The model was adjusted for age, gender and total family income (Kuwaiti Dinars/ month)

^b^ Diagnosed as per ISAAC criteria

## Discussion

This cross-sectional study assessed the prevalence of self-reported asthma and examined ETS exposure, active tobacco smoking along with demographic, lifestyle and behavioural characteristics in relation to self-reported asthma status among adolescents in Hawally Governorate, Kuwait. The prevalence estimates of self-reported asthma and physician-diagnosed asthma were 20.5% and 16.4% respectively. The prevalence estimates for self-reported asthma is higher than the figures reported by earlier ISAAC studies conducted in Kuwait during 1995–1996 (16.8%) [8], and 2001–2002 (15.6%) [9]. In contrast, the prevalence estimate of physician-diagnosed asthma (16.4%) in this study is largely in agreement with the estimates from the 1995–1996 (16.8%) and 2001–2002 (15.6%) ISAAC studies in Kuwait [8, 9]. The estimate of self-reported asthma in this study is also greater than an estimate (14.6%) reported in a recent study among university students in Kuwait [15]. Additionally, prevalence estimate (20.5%) of self-reported asthma among high school students in this study was fairly comparable with the estimates reported among high-school students in Saudi Arabia (18.5%) [19], Lebanon (19.5%) [12], Virginia, USA (16%) [20], Lima, Peru (16.7%) [21], and much higher than a prevalence (10.7%) estimated among 6–15 years old 23,044 Japanese students based on ISAAC criteria [22]. Across Latin American countries, a wide variation in one-year prevalence of self-reported asthma diagnosed based on ISAAC criteria was recorded. This variation ranged from 6 to 28% among adolescents (13–14 years) and 7–27% among children (6–7 years) [23], with some countries registered higher prevalence estimates than the one in the present study. Furthermore, in the present study, 12-month prevalence of wheezing was 20.1%, which is higher than the 12-month prevalence estimates reported in earlier ISAAC studies conducted in Kuwait during 1995–1996 (16.1%) [8], and 2001–2002 (7.6%) [9]. Additionally, the 12-month prevalence of wheezing (20.1%) in the current study is also higher than the global estimate of 14.8% among adolescents aged 13–14 years [7]. Evaluation of worldwide trends in the prevalence of asthma symptoms based on ISAAC Phase III study has shown that while there was little change in the overall prevalence of current wheeze, the proportions of the children reported to have had asthma increased significantly, possibly reflecting greater awareness of this condition and/or changes in diagnostic practice. However, it was recognized that the increases in asthma symptoms prevalence in Africa, Latin America and parts of Asia indicate that the global burden of asthma is continuing to rise, but the global prevalence differences are lessening [24]. Hence with 20.5% prevalence estimate of self-reported asthma, Kuwait can be bracketed with the group of countries with high prevalence of self-reported asthma among adolescents. These differences in the self-reported asthma prevalence could possibly be due to varying distributions of underlying contributing factors such as populations’ genetics, dietary habits, microbial exposure, economic status, indoor or outdoor environment, climatic variation, and disease awareness [25, 26]. It has been argued that the global increases in asthma prevalence appear to include both allergic and non-allergic asthma which highlights the importance of considering the heterogeneity of asthma with different phenotypes having different pathophysiologic mechanisms [27]. Therefore, monitoring of adolescents’ respiratory disorders including asthma and identification of underlying factors in various geographical regions is warranted to alleviate the burden of asthma and related complications.

Multivariable log-binomial regression model showed that compared to non-smokers, current smoker adolescents were significantly more likely to be asthmatic. This finding is consistent with the reports from Britain [28], Argentina [29], South Korea [30, 31], wherein smoker adolescents reportedly were at greater risk for current self-reported asthma. Relatively a recent study also found 70% increased asthma risk among smokers than non-smokers young adults enrolled in a public sector university in Kuwait [15]. Thus, cconcerted efforts at high school-level to increase the awareness regarding deleterious effects of tobacco smoking may help in reducing tobacco consumption among adolescents.

Final multivariable log-binomial regression model also revealed that the adolescents were significantly more likely to be asthmatic if they have had ETS exposure at home. This finding is in agreement with the results of earlier cross-sectional studies undertaken in various regions across the globe using ISAAC methodology [29, 32]. These reports showed that adolescents were more likely to develop asthma if either or both parents were smokers compared to non-smoking parents [11, 29, 32, 33]. Another cross-sectional study from Spain showed that parental smoking was associated with a higher prevalence of all forms of asthma in the adolescents population, particularly if mother or both parents smoked [34]. In Mexico, a case-control study showed that adolescents with asthma nearly were twice as likely to report one or more smokers at home as those without asthma [35]. In Sweden, a longitudinal population-based cohort study of children recruited at birth and followed through childhood and adolescence demonstrated 68% increased risk of asthma among children up to 16 years of age born to heavy (≥ 10 cigarettes /d) smoking compared to non-smoking mothers [36]. To demonstrate the consistency of relationship between tobacco smoking and the asthma risk across the globe, Mitchell and colleagues analyzed the ISAAC programme Phase III data on the 6–7 year age group (220,407 children from 75 centres in 32 countries) and 13–14 year age group (350,654 adolescents from 118 centres in 53 countries) and reported a significant association between current maternal smoking and current asthma symptoms. This association was held across all nine world regions covered by the study including Eastern Mediterranean region. Moreover the investigators showed a dose-response relationship between severe asthma symptoms and number of cigarettes smoked per day by both the parents [11]. Thus, cumulated anecdotal evidence on the link between ETS exposure and asthma risk among adolescents warned the causal relationship [36–38]. Furthermore, it has been argued that while substantial advances in asthma treatment were made in past several decades, it is evident that in the future, asthma is expected to create a considerable strain on a large population with inadequate access to health care [27].

Globally, Kuwait ranks high in regard to tobacco smoking among adult men (23.5%) and women (5.5%) [39]. Furthermore, a global school-based student health survey report revealed that 60% of the students in Kuwait reported that people smoked in their presence on one or more days during the past seven days [40]. These reports reflect that Kuwaiti adolescents not only get exposed to ETS at homes but also outside the homes. Though Kuwait has enacted a law forbidding tobacco smoking at public places such as restaurants, shopping malls, public transport, public offices etc., yet this law is not strictly enforced. Resultantly frequent violation of this law prohibiting tobacco smoking at public places occurs [41].

Currently ETS exposure is the only avoidable risk factor for which considerable evidence of an etiologic link with asthma among adolescents is available with population attributable risk ranging from 2 to 20% depending upon the level of maternal and paternal smoking [11]. Therefore, voluntary household smoking bans may substantially minimize the ETS exposure among adolescents at home. Furthermore, such a restriction may inculcate an antismoking attitude and prevent smoking initiation among adolescents. Additionally, strict imposition of existing law forbidding smoking at public places and heavy penalty to those who show disrespect to this law is warranted. Such efforts are likely to pay dividends in terms of reduction in ETS exposure and associated asthma risk and other smoking related morbidities in this and other similar settings as have been shown in other populations [34, 42].

There are some notable strengths of this study. *First*, the study sample comprised participants who were homogenous regarding age. *Second*, the use of gender-stratified sampling allowed enrollment of an almost equal number of male and female participants. *Final*, the use of the standardized and validated ISAAC instrument for the outcome assessment facilitated the comparison of the study results with that of other local, regional and international studies. This study has some limitations which should be taken into account in interpretation of the results. *First*, this was a cross-sectional study and this design has inherent limitation in establishing temporal association between the evaluated exposures including self-smoking history, ETS exposure at home and self-reported asthma. *Second*, past one-year data were collected with chances of recall bias in completing the self-administered study instrument*.* However, one-year period presumably was not long enough to severely hamper the recall of the events. *Third,* we estimated the asthma prevalence for the past 12 months and if the children grew out of asthma, which they might have early on in their life then such cases were likely to be missed out as asthmatic in this evaluation. This might have led somewhat underestimation of self-reported asthma prevalence. However, due to a chronic nature of the disease, the number of such adolescents is likely to be very small and indeed might have negligible influence on the study results. *Fourth,* we enrolled the adolescents in the study as a sample of convenience, therefore, generalizability of the results to the other adolescents in the country and beyond should be exercised with care. However, we do not have any reason to believe that the adolescents in the study were any different from those at large in the population. Additionally, though our sample was statistically non-representative but was typical of Kuwaiti adolescents in the population at large. *Final*, the responses to the questions on asthma status were self-reported and not validated by more objective measurements. However, the ISAAC questionnaire used in this evaluation has been validated in multiple languages in different countries including Kuwait with high sensitivity and specificity [8, 9, 11].

## Conclusion

We recorded high prevalence of ETS exposure at home, a high prevalence of self-reported asthma and identified ETS exposure at home and being a current smoker as strong risk factors for self-reported asthma among adolescents. Therefore, focused education to minimize the exposure to tobacco smoke through active and/ or passive modes may alleviate the burden of asthma and other tobacco smoking-related morbidities among adolescents in this and other similar settings. If implemented, future studies may look at the impact of such interventions.

## Data Availability

The data collected and analyzed are included in this manuscript and can be made available on reasonable request to the corresponding author.

## Abbreviations

ETS: Environmental Tobacco Smoke
ISAAC: International Study of Asthma and Allergies in Childhood
SD: Standard Deviation
PR: Prevalence Ratio
CI: Confidence Interval
